# Peripheral Retinal Degenerations and Idiopathic Epiretinal Membrane: Analysis with Ultra-Wide-Field Scanning Laser Ophthalmoscopy

**DOI:** 10.3390/jcm10173876

**Published:** 2021-08-28

**Authors:** Klaudia Ulfik-Dembska, Sławomir Teper, Michał Dembski, Anna Nowińska, Edward Wylęgała

**Affiliations:** 1 Faculty of Medical Sciences in Zabrze, Medical University of Silesia, Poniatowskiego 15, 40-760 Katowice, Poland; slawomir.teper@gmail.com (S.T.); michau32@gmail.com (M.D.); anna.nowinska@sum.edu.pl (A.N.); rekroz@sum.edu.pl (E.W.); 2 Clinical Department of Ophthalmology, District Railway Hospital, Panewnicka 65, 40-760 Katowice, Poland

**Keywords:** idiopathic epiretinal membrane, ultra-wide-field laser scanning ophthalmoscopy, peripheral retinal abnormalities, Goldmann three-mirror contact lens

## Abstract

Background: The present study examined the relationships among retinal structure, peripheral retinal abnormalities, and epiretinal membrane (ERM) and explored the utility of ultra-wide-field laser scanning ophthalmoscopy in idiopathic ERM assessment. Methods: The study sample comprised 276 eyes of 276 patients. Ultra-wide field fundus imaging was performed without mydriasis using Optos California. Each patient underwent a Goldmann three-mirror contact lens fundus examination. Results: Ultra-wide field laser scanning ophthalmoscopy revealed peripheral retinal degeneration in 84 (54.54%) eyes in the ERM and in 28 (22.95%) eyes in the control group. Goldmann three-mirror contact lens examination revealed peripheral retinal degeneration in 96 (62.33%) eyes in the ERM group and 42 (34.42%) eyes in the control group. Ultra-wide field ophthalmoscopy enabled the detection of nearly 87% of all peripheral retinal lesions in patients with ERM, but it cannot replace fundus examination with a Goldmann triple mirror or ophthalmoscopy with scleral indentation. Conclusions: In most patients, idiopathic ERM coexisted with changes in the peripheral retina. Some of these changes promote retinal detachment. Thus, surgeons should consider the risk of retinal tear during vitrectomy, which increases the scope of surgery and may adversely affect prognosis. Although ultra-wide field imaging is a valuable diagnostic method, it is not a substitute for Goldmann three-mirror contact lens fundus examination or ophthalmoscopy with scleral indentation.

## 1. Introduction

The epiretinal membrane (ERM) is a thin layer of fibrous tissue forming on the inner surface of the central retina. It causes metamorphopsia, monocular diplopia, and vision loss [1]. The exact etiology of this condition remains unknown, but it can be idiopathic or secondary to other ocular diseases, trauma, or previous intraocular operation. Electron microscopy has revealed the involvement of glial cells, retinal pigment epithelial cells, fibrocytes, myofibroblasts, and fibrous astrocytes in ERM [2]. Cells that infiltrate the retinal surface to form ERM may originate from lesions at the far periphery of the retina. The incidence of idiopathic ERM ranges from 2% in patients below 60 years of age to 12–20% in those above 70 years of age [3]. However, up to 90% patients may remain asymptomatic [4]. Although ERM is typically detected through fundus examination, the use of optical coherence tomography (OCT) has increased detection sensitivity [5]. The symptoms are highly subjective, and the deterioration of visual acuity is often slight. In this light, more objective morphological factors indicating the severity of the disease must be identified. These factors must be directly related to retinal function and symptom severity. ERM leads to changes in retinal morphology, which is evident in fundus examination.

Asymptomatic retinal breaks occur in approximately 7% of patients over age 40, and lattice degeneration is present in approximately 8% of the general population. Since retinal breaks cause retinal detachment and lattice degeneration is associated with approximately 30% of retinal detachments, prophylactic treatment of these lesions has sometimes been recommended [6]. Lattice degeneration of the retina is the most important vitreoretinal abnormality predisposing to retinal detachment (RD) [7]. The studies have shown that the prevalence of lattice in patients with bilateral RDs is higher than in patients with unilateral RDs, perhaps indicating that lattice predisposes to bilateral RDs [8,9].

Goldmann three-mirror contact lens examination remains the standard for peripheral retinal assessment. Likewise, indirect ophthalmoscopy with sclera indentation provides a complete picture of the fundus. Currently, ultra-wide field fundus imaging is frequently being infused in clinical practice. Fundus imaging covering ≥100° of the retina is considered ultra-wide field examination [10]. Optos California (Optos PLC, Dunfermline, UK), the pioneer ultra-wide field retinal imaging system, uses a scanning laser ophthalmoscope to obtain retinal images without mydriasis. This imaging system was designed to cover up to 200° of the retina within a single image (>80% of the retina) [11]. The fundus photograph obtained using Optos is formed by a combination of monochromatic red and green scanning laser images. Color images are captured in pseudocolor using bicolor laser [red (633 nm) and green (532 nm)]. Fundus autofluorescence can also be detected using a green laser (532 nm) for excitation and an emission filter (570–780 nm) [12]. A semi-realistic biocolor Optos fundus image is often different from a real color image. To the best of our knowledge, no previous study has analyzed multimodal imaging data combining fundus scans obtained using ultra-wide field retinal imaging and Goldmann three-mirror contact lens fundus examination in patients with idiopathic ERM.

The aim of the present study was to examine the relationships among retinal structure, peripheral retinal abnormalities, and ERM and to explore the utility of ultra-wide field retinal imaging for ERM assessment.

## 2. Materials and Methods

### 2.1. Study Participants

This randomized prospective observational study was performed with adherence to the tenets of the *Declaration of Helsinki* and was approved by the Bioethical Commission at the Medical University of Silesia in Katowice, Poland (KNW/0022/KB1/86/18). The participants were informed of the purpose, nature, and method of research. After providing written informed consent, patients were qualified for the research project. The study sample comprised 276 eyes of 276 patients.

The inclusion criteria were as follows: consent to participate in the study, presence of idiopathic ERM, and age > 18 years. The exclusion criteria were as follows: history of uveitis, penetrating eye injuries, pars plana vitrectomy, high myopia, or diabetes; media opacities resulting in low-quality imaging; obstacles in obtaining sufficient pupil dilation; and pregnancy.

### 2.2. Examination Procedure

After obtaining signed informed consent, detailed clinical history was collected for all patients. The patients underwent comprehensive ophthalmologic examination using a slit lamp (SL 990 Digital Version; CSO; Firenze, Italy), measurement of the best corrected visual acuity (BCVA), tonometry (Goldmann applanation tonometry), color, and autofluorescence fundus photography using an ultra-wide field imaging system. Stereoscopic fundus examination was performed always by the one trained retina expert using a Goldmann three-mirror contact lens with mydriasis. BCVA was measured using the Snellen visual acuity charts. Data on age, sex, previous and current ophthalmic history, and lens status were collected.

**Figure 1:**
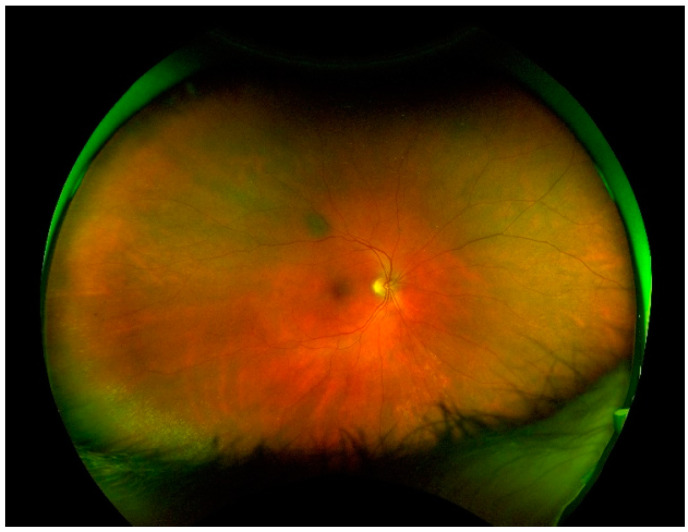
Color image of the fundus obtained using ultra-wide field laser scanning ophthalmoscopy in a patient with epiretinal membrane and exhibiting benign peripheral lesions of the retina and choroidal nevus.

**Figure 2:**
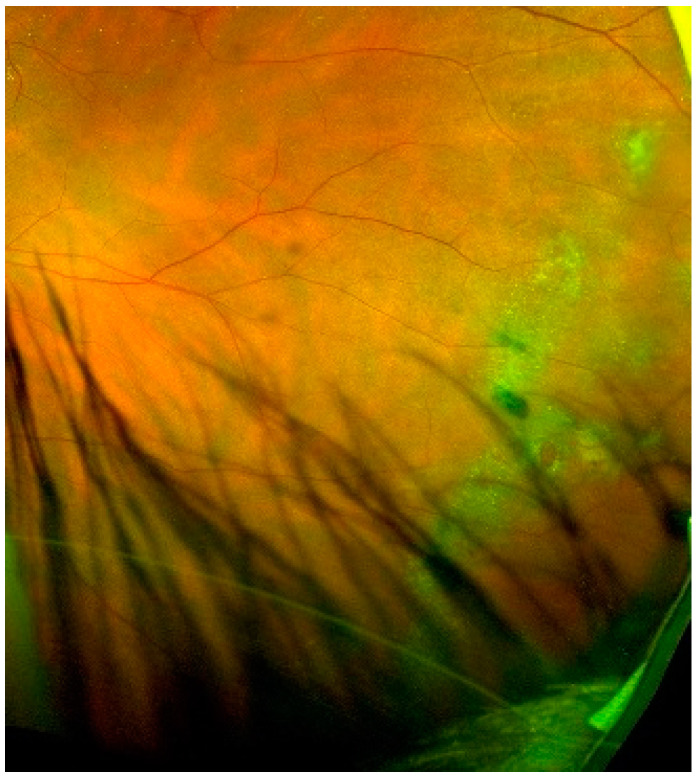
Color image of the fundus obtained using the ultra-wide field laser scanning ophthalmoscopy in a patient with ERM and exhibiting “snail track” lesions of the retina.

**Figure 3:**
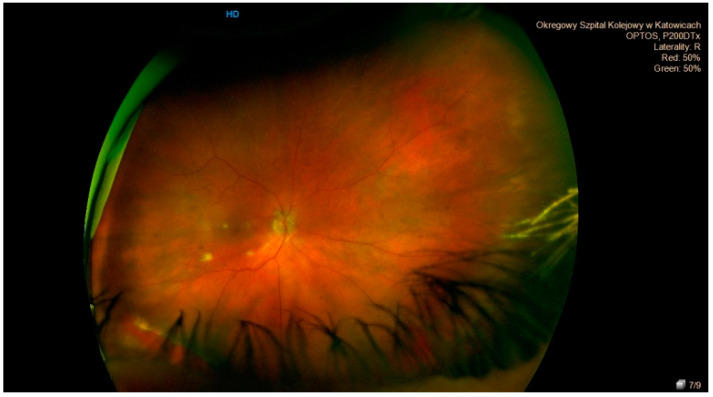
Retinal break—a severe degenerative lesion of the peripheral retina.

**Figure 4:**
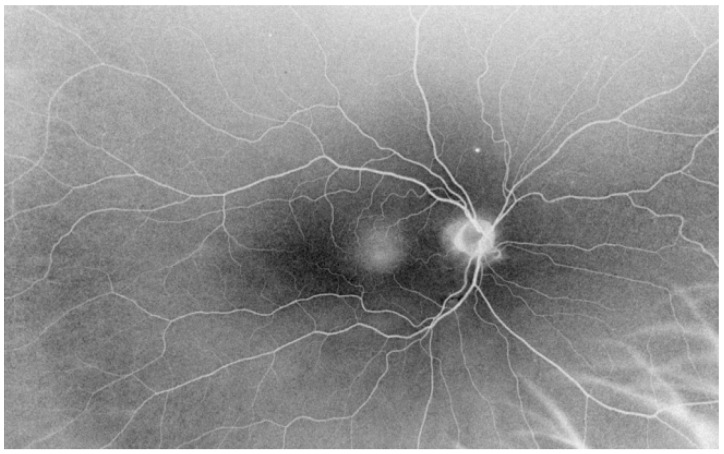
Black-and-white image of the fundus obtained using ultra-wide field laser scanning ophthalmoscopy in a patient with epiretinal membrane.

Ultra-wide field fundus imaging was performed without mydriasis using Optos California (Optos PLC). The images were centered on the macula, steered in four directions, and finally exported for analysis. All images obtained using ultra-wide-angle laser ophthalmoscopy were imported as color and black-and-white photographs in the JGP format (9600 × 4326 pixels) (Figure 1, Figure 2, Figure 3 and Figure 4).

### 2.3. Statistical Analysis

Statistical analysis was performed using Statistica version 13.1 (TIBCO Software Inc., Palo Alto, CA, USA) with Pandas and Penguin statistical packages for Python. A *p*-value less than 0.05 was considered significant, considering multiple testing for interpretation. The normality of the variable distribution was examined using the Shapiro–Wilk test. Statistical significance was calculated using a *t*-test for parametric variables and Mann Whitney U-test for non-parametric variables. For further analysis, non-parametric Spearman’s correlation coefficients were used to determine statistical dependence.

## 3. Results

A total of 154 eyes with ERM and 122 normal (control) eyes were analyzed. If ERM was present in both eyes, only one eye of each participant was randomly selected for posterior segment imaging. In control patients, only one eye was randomly selected.

The mean age of patients in the ERM group was 71.41 ± 8.19  (range 53–86) years and that of patients in the control group was 59.26 ± 21.21 (range 21–83) years. A total of 112 (72.72%) eyes with ERM were females and 42 (27.27%) were males. Moreover, 68 (55.73%) eyes in the control group were females and 54 (44.26%) were males. In the ERM group, 102 (66.23%) eyes were phakic and 52 (33.76%) were pseudophakic. In the control group, 96 (78.68%) eyes were phakic and 26 (21.31%) were pseudophakic. Patients with high myopia were excluded from the initial stage of the study. The mean axial length of the eyeball in the control group was 23.43 mm, and in the ERM group, it was 23.51 mm (p = 0.34).

The mean visual acuity was 0.204 logMAR in the ERM group and 0.079 logMAR in the control group. The mean intraocular pressure was 14.31 ± 2.82 (range 11–21) mmHg in the ERM group and 14.52 ± 2.43 (range 11–19) mmHg in the control group.

Goldmann three-mirror contact lens examination revealed peripheral retinal degeneration in 96 (62.33%) eyes in the ERM group and 42 (34.42%) eyes in the control group. The results are summarized in Table 1.

Ultra-wide field laser scanning ophthalmoscopy revealed peripheral retinal degeneration in 84 (54.54%) eyes in the ERM and in 28 (22.95%) eyes in the control group (Table 2). These results indicate that peripheral degeneration is more common in patients with ERM (p = 0.03). The most common change in both groups was paving stone degeneration. Goldmann three-mirror contact lens examination is characterized by greater detectability of peripheral degenerations, particularly in the upper and lower quadrants. Goldmann three-mirror contact lens examination revealed 4% lesions in the nasal quadrant, 30% in the temporal quadrant, 28%in the lower quadrant, and 38% in the upper quadrant. Ultra-wide field laser scanning ophthalmoscopy revealed 4% lesions in the nasal quadrant, 49% in the temporal quadrant, 25% in the lower quadrant, and 22% in the upper quadrant; the latter method detected fewer peripheral degenerations in the upper and lower quadrants, which was probably because of the presence of upper and lower eyelashes and drooping eyelids. Ultra-wide field laser scanning ophthalmoscopy detected approximately 87% of the peripheral lesions compared with Goldmann three-mirror contact lens examination. Statistical comparison was performed between two groups: ERM group and control group (Table 1 and Table 2). The table shows p-values for the Mann–Whitney U-test. Statistically significant differences are distinguished. Statistically significant differences were found for paving stones and retinal break in the group examined using Goldmann three-mirror lens and in the group examined using ultra-wide field laser opthalmoscopy.

## 4. Discussion

The present study demonstrated a significant association between the presence of ERM and peripheral degenerative changes in the retina. To our knowledge, the presented study is the most extensive research study investigating the relationship between idiopathic epiretinal membrane and peripheral degenerations.

ERM comprises a sheet of fibrotic tissues, which can vary in thickness from a single layer of collagen with interspersed cells to a thicker, manifold fibrous cell layer. Although the pathogenesis of ERM has not been fully elucidated this far, the formation and progression of ERM can be regarded as fibrotic processes, because the pathological findings include increased protein deposition and membrane contraction in which myofibroblasts play crucial roles [13,14]. Specific cells (e.g., glial cells, neurites, retinal pigment epithelial cells, fibrocytes, and Müller cells) may migrate from the retina through small defects in the inner limiting membrane (ILM) and may subsequently emerge on the retinal surface through peripheral degenerative changes. In our study, patients in the control group were relatively younger than those with ERM, which proves that the incidence of the idiopathic retinal membrane increases with age [15,16]. Visual acuity was statistically better in the control group than in the ERM group. Furthermore, other recent reports have described the photoreceptor cone outer segment tips to be highly associated with BCVA in the ERM group [16].

According to the literature, peripheral drusen, reticular pigmentary change, and paving stone degeneration occurred significantly more frequently in patients with AMD than in those without it [17,18]. This supports the notion that the disease is pan-retinal and not limited exclusively to the macula. A similar process probably occurs in eyes with ERM. Retinal tears and holes unassociated with acute symptoms and lattice degeneration are significantly less likely to be the sites of retinal breaks, which are responsible for subsequent retinal detachment [19]. Lattice degeneration is the most important of all clinically distinct entities that affect the peripheral fundus and which are related to retinal detachment [20,21]. The lattice degenerations, snail track degenerations, and retinal breaks can lead to retinal detachment [22]. Peripheral degenerative changes may serve as the site of migration to the surface of the retina for cells forming the membrane. Interestingly, in our study in patients with ERM, severe peripheral retinal degeneration was more common, which may result in retinal detachment. The present study revealed numerous degenerative changes, such as retinal break and retinoschisis, but most patients were asymptomatic. Both Goldmann three-mirror contact lens examination and ultra-wide field laser scanning ophthalmoscopy revealed more peripheral degenerations in the ERM group than in the control group of this study. The percentage of lattice degenerations, snail track degenerations, retinal breaks, and paving stone degenerations was high in the ERM group. On the other hand, the fact is that the incidence of paving stone type degeneration increases with age [23]. In our study, ERM patients were relatively older than the control group.

As expected, three-mirror contact lens examination showed greater sensitivity of detection for peripheral degenerations than did ultra-wide field ophthalmoscopy. This may be explained by the presence of upper and lower eyelashes, obscuring the degenerations of the lower and upper retina located at the far periphery. In a comparative study of peripheral degenerations in patients with high myopia, Liu et al. [24] showed comparable sensitivities of Goldmann three-mirror contact lens examination and ultra-wide field ophthalmoscopy. Ultra-wide field ophthalmoscopy is characterized by a range of 200°, albeit only in the temporal quadrant. In the present study, we confirmed that ultra-wide field ophthalmoscopy may not be suitable for detecting peripheral retinal lesions, particularly those in the lower and upper quadrants. The publications report that sometimes, despite very careful examinations of the patient with a slit lamp and a Goldmann three mirror, some of the degenerative retinas remain undetected [8]. This proves that the number of peripheral degenerations detected by the Goldmann three-mirror test also cannot be treated as the final number of degenerations present.

Given the increasing use of this imaging modality for screening purposes, its limitations must be taken into account.

The study is not without certain limitations. The limitations of this study include the relatively small sample size (154 eyes with ERM and 122 control eyes). We will make an effort to increase the study group size for further analysis. Another limitation of the study is the lack of the examinations with sclera indentation. No method guarantees 100% detection of peripheral retinal degeneration. The next limitation is only one examination of each patient. The study requires further research.

## 5. Conclusions

1. In most people, ERM coexists with peripheral retinal changes.
2. Some of these changes promote retinal detachment. Thus, surgeons should consider the risk of retinal tear during vitrectomy, which increases the scope of surgery and may adversely affect prognosis. According to the results obtained in our study, we strongly suggest examining patients before the vitrectomy (ILM peeling) procedure with a Goldmann triple mirror.
3. Ultra-wide field ophthalmoscopy is a valuable diagnostic method. It enabled the detection of nearly 87% of all peripheral retinal lesions in patients with ERM, but it cannot replace fundus examination with a Goldmann triple mirror or ophthalmoscopy with scleral indentation.

## Figures and Tables

**Table 1:** Peripheral retinal changes in patients without (control) and with epiretinal membrane (ERM) detected using Goldmann three-mirror contact lens examination.

	Lattice Degeneration	Snail Track Degeneration	Paving Stone Degeneration	Retinoschisis	Microcystoid Degeneration	Retinal Break
**ERM group**	14 (14.58%)	12 (12.5%)	33 (34.37%)	7 (7.29%)	11 (11.45%)	19 (19.79%)
**Control group**	7 (16.66%)	6 (14.28%)	15 (35.71%)	5 (11.90%)	5 (11.90%)	4 (9.52%)
**Statistical comparison**	*p* = 0.29	*p* = 0.33	*p* = 0.047	*p* = 0.85	*p* = 0.28	*p* = 0.006

**Table 2:** Peripheral retinal changes in patients with epiretinal membrane and in the control group using UWF laser opthalmoscopy.

	Lattice Degeneration	Snail Track Degeneration	Paving Stone Degeneration	Retinoschisis	Microcystoid Degeneration	Retinal Break
**ERM group**	11 (13.0%)	9 (10.7%)	31 (36.90%)	5 (5.95%)	10 (11.90%)	18 (21.42%)
**Control group**	4 (14.28%)	2 (7.14%)	11 (39.28%)	3 (10.71%)	5 (17.85%)	4 (14.28%)
**Statistical comparison**	*p* = 0.16	*p* = 0.077	*p* = 0.01	*p* = 0.40	*p* = 0.38	*p* = 0.01

## Data Availability

The data presented in this study are available in Chair and Clinical Department of Ophthalmol-ogy, Faculty of Medical Sciences in Zabrze, Medical University of Silesia in Katowice, 40-055 Ka-towice, Poland.

## Abbreviations

ERM	Epiretinal Membrane
OCT	Optical Coherence Tomography
OCT-A	Optical Coherence Tomography Angiography
UWFP	Ultra-Wide Field Laser Opthalmoscopy
BCVA	Best Corrected Visual Acuity
ILM	Internal Limiting Membrane
AMD	Age-Related Macular Degeneration
DR	Diabetic Retinopathy
RD	Retinal Detachment
